# Commentary: The Impact of Neuroimmune Alterations in Autism Spectrum Disorder

**DOI:** 10.3389/fpsyt.2015.00145

**Published:** 2015-10-08

**Authors:** Dario Siniscalco

**Affiliations:** ^1^Department of Experimental Medicine, Second University of Naples, Naples, Italy; ^2^Centre for Autism – La Forza del Silenzio, Caserta, Italy; ^3^Cancellautismo – No Profit Association for Autism Care, Florence, Italy

**Keywords:** autism, neuroimmune interactions, epigenetics, monocytes, cytokines

The dramatic increasing prevalence of autism spectrum disorders (ASDs) (1), together with the influence on the quality of life and the lifetime societal cost of caring, has called for newest research on both the development of these diseases and the therapeutic options. Nowadays, it is well recognized that multifactorial and polygenic features (complex combination of genetic, epigenetic, and environmental interactions) characterize ASDs (2). Prenatal immune alterations and early inflammatory processes could be the autism etiological events. The authors Gottfried et al. (3) in this hypothesis-and-theory article discuss the recent findings in autism discovery. Starting from a brief historical way on autism development, the main topic of the article is to focus on the state-of-the-art of the novel findings in autism studies. The authors rightly highlight the newest challenging frontier of autism research: the neuroimmune axis alterations. These alterations are first evident in the cells early responsible for immune responses, as they are the precursors for macrophages, dendritic, and microglial cells: monocytes or peripheral blood mononuclear cells (PBMCs). These cells show strong dysfunctions in ASD children and are committed to a pro-inflammatory state, which in turn result in long-term immune alterations (4). In ASDs, altered PBMCs are responsible for elevated pro-inflammatory cytokine production. The up-regulation of inflammatory cytokines is also reflected in brain centers of autistic patients (5): the consequences are the induction of blood–brain barrier (the immunological interface between peripheral immune system and central nervous system) disruption. Changes in BBB permeability directly influence neural plasticity, connectivity and function, triggering impairments in social interaction, communication, and behavior (3). Immunological abnormalities also influence the gastrointestinal system and the microglial innate immune cells of the central nervous system (6). The authors also discuss the role of autoimmunity in the pathogenesis of autism. Familial or virus/bacteria-infected autoimmunity could be a risk factor for autism. Even if the exact cellular and molecular pathways responsible for the induction of neuroimmune alterations are still to be further clarify, a complex interaction among epigenetic and environmental risk factors (7) could trigger the neuroimmune abnormalities, such as abnormal neuron and glia responses.

Taken together, these autism-associated neuroimmune changes could help in identifying novel therapeutic target for a better future management of ASDs.

## Conflict of Interest Statement

The author declares that the research was conducted in the absence of any commercial or financial relationships that could be construed as a potential conflict of interest.
